# Reduction in oxidatively generated DNA damage following smoking cessation

**DOI:** 10.1186/1617-9625-9-5

**Published:** 2011-05-12

**Authors:** Harold C Box, Richard J O'Connor, Helen B Patrzyc, Herbert Iijima, Jean B Dawidzik, Harold G Freund, Edwin E Budzinski, K Michael Cummings, Martin C Mahoney

**Affiliations:** 1 Roswell Park Cancer Institute, Elm and Carlton Streets, Buffalo, NY 14263, USA

## Abstract

**Background:**

Cigarette smoking is a known cause of cancer, and cancer may be in part due to effects of oxidative stress. However, whether smoking cessation reverses oxidatively induced DNA damage unclear. The current study sought to examine the extent to which three DNA lesions showed significant reductions after participants quit smoking.

**Methods:**

Participants (n = 19) in this study were recruited from an ongoing 16-week smoking cessation clinical trial and provided blood samples from which leukocyte DNA was extracted and assessed for 3 DNA lesions (thymine glycol modification [d(T^g^pA)]; formamide breakdown of pyrimidine bases [d(T^g^pA)]; 8-oxo-7,8-dihydroguanine [d(G^h^)]) via liquid chromatography tandem mass spectrometry (LC-MS/MS). Change in lesions over time was assessed using generalized estimating equations, controlling for gender, age, and treatment condition.

**Results:**

Overall time effects for the d(T^g^pA) (χ^2^(3) = 8.068, p < 0.045), d(P^f^pA) (χ^2^(3) = 8.477, p < 0.037), and d(G^h^) (χ^2^(3) = 37.599, p < 0.001) lesions were seen, indicating levels of each decreased significantly after CO-confirmed smoking cessation. The d(T^g^pA) and d(P^f^pA) lesions show relatively greater rebound at Week 16 compared to the d(G^h^) lesion (88% of baseline for d(T^g^pA), 64% of baseline for d(P^f^pA), vs 46% of baseline for d(G^h^)).

**Conclusions:**

Overall, results from this analysis suggest that cigarette smoking contributes to oxidatively induced DNA damage, and that smoking cessation appears to reduce levels of specific damage markers between 30-50 percent in the short term. Future research may shed light on the broader array of oxidative damage influenced by smoking and over longer durations of abstinence, to provide further insights into mechanisms underlying carcinogenesis.

## Introduction

A commonality in the etiology of cancers may be DNA damage arising from oxidative stress [1,2]. There are multiple reasons to associate oxidative stress with cancer. Oxidative DNA damage can cause transcription errors, replication errors, and genomic instability, which are all associated with carcinogenesis [3-7]. Over 100 oxidative DNA damage products are known, and reactive oxygen species (ROS) can induce DNA breaks, purine, pyrimidine, or deoxyribose lesions, and even cross links among these [5].

Oxidative stress in cells and organisms is caused by the presence of ROS, including hydroxyl radicals, superperoxide, hydrogen peroxide, and singlet oxygen. Cells or organisms having an inordinately high level of ROS are said to be under oxidative stress. ROS are generated inadvertently in the mitochondria of all cells concomitant with the synthesis of ATP. ROS arise due to oxygen that escapes complete reduction. Other in-vivo sources of ROS include inflammatory responses and detoxification processes. Cigarette smoking is an important cause of cancer [8] and it is well established that tobacco smoke contains thousands of chemicals and causes inflammation. It is also known that ROS are generated during the combustion of tobacco products [9-11]. Cancer risk associated with oxidative stress may be explained in that ROS can cause oxidative DNA damage that lead to mutations that lead to cancer.

The connection between environmental exposures like cigarette smoking and cancer may be better understood by characterizing the DNA damage involved in the carcinogenic process. Prior work in examining environmental sources of oxidative damage has generally focused on the 8-oxo-7,8-dihydroguanine [d(G^h^)] lesion. Findings on cigarette smoke exposure have been decidedly mixed [10]. Priemé and colleagues [12] reported a decrease of about 20% in d(G^h^) among those quitting smoking up to 26 weeks. Lodovici et al., [13] as well as Asami et al., [14] reported a significantly lower mean value of d(G^h^) in leukocyte DNA of non-smokers compared with smokers while Nia et al., [15] and Van Zeeland et al. [16] reported a lower average value for d(G^h^) in lymphocyte DNA of smokers compared with non-smokers. Lodovici et al., [13] also demonstrated that d(G^h^) was elevated in those exposed to secondhand smoke, similar to an earlier finding by Howard et al. [17] showing elevated levels in those occupationally exposed to SHS. However, Collier and colleagues [18] have shown that men and women differed in their oxidative damage levels due to SHS exposure, with a more prominent dose-response effect seen in men.

**Figure 1:**
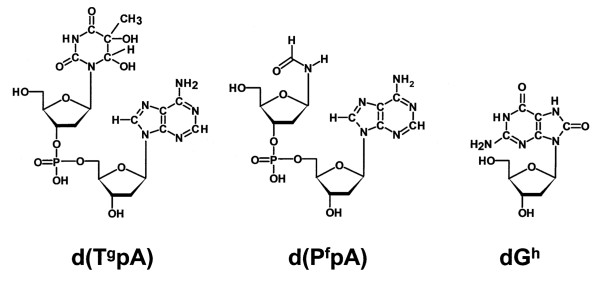
**DNA dimer modifications under investigation**.

An issue with the existing literature is the reliance on the d(G^h^) lesion as the primary indicator of oxidatively-induced DNA damage from cigarette smoking. This base modification is not a significant product of deoxyguanosine exposed in vitro to hydroxyl radicals [19]. In addition, the facile oxidation of guanine leads to artifactual production of d(G^h^). A preferable approach may be to examine base modifications that unequivocally can be associated with hydroxyl radical activity. Modern mass spectrometry now makes it feasible to measure the levels of multiple oxidatively-induced DNA lesions simultaneously. Two such alternative base modifications are a) the glycol modification of thymine [d(T^g^pA)], and b) the formamide breakdown product of pyrimidine bases [d(P^f^pA)]. The structures of these modifications are shown in Figure 1 in the form they are measured. Included in Figure 1 is the structure of d(G^h^), the DNA modification most often used as an indicator of oxidative stress.

The current study sought to examine the extent to which these three DNA lesions would show measurable change upon cessation of smoking in a longitudinal fashion. Our study is notable in two respects: longitudinal measurements were a component of an on-going smoking cessation study and study subjects served as their own controls.

## Methods

### Experimental Procedures

**Figure 2:**
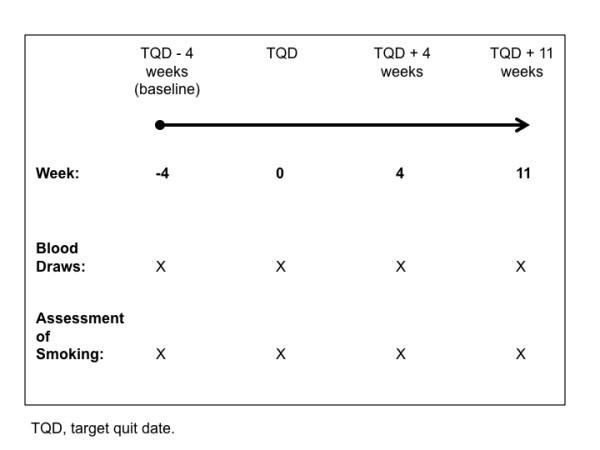
**Study timeline**.

Participants (n = 19) in this study were recruited from an ongoing 16-week smoking cessation clinical trial using varenicline. Criteria for inclusion were smoking at least 15 cigarettes per day, general good health, and willingness to make a quit attempt. Persons were excluded if they currently used tobacco products other than cigarettes; were using smoking cessation drugs at time of enrollment (e.g., varenicline, bupropion, nicotine); had a serious medical or mental health condition in the past year; abused alcohol or other drugs; or were pregnant or planning to become pregnant. Eligible participants received either medication (varenicline) or a placebo as part of a double-blind cessation study. Both self-reported tobacco use and measured breath carbon monoxide (CO) levels were used to determine tobacco use status at each visit. Blood samples were obtained from volunteer donors at baseline (4 weeks prior to target quit ), on the target quit date (Study Week 0), 4 weeks following target quit date (Study Week 4), and 11 weeks after target quit date (Study Week 11). (see Figure 2) Participants received $25 remuneration for each blood sample provided. The study protocol was reviewed and approved by the Roswell Park Cancer Institute Institutional Review Board. All participants provided written informed consent, and provision of blood samples was an optional component of the clinical trial.

### Analytical Procedures

Blood samples were drawn with EDTA as anticoagulant, centrifuged, and the buffy coat collected. DNA was extracted from the cells using a kit designed to minimize spurious oxidation reactions (ZeptoMetrix, Inc., Buffalo, NY). The kit employs chaotropic precipitation of the DNA together with desferol in the extraction procedure. One hundred μg of DNA was hydrolyzed and dephosphorylated using nuclease P1 and alkaline phosphatase. A solution containing 15 μl sodium acetate buffer (0.25 M, pH 5.2), 50 μl 3.0 mM Zn Cl_2_, 50 μL water and 1.0 U nuclease P1 (Sigma N8630) together with the DNA and isotopically labeled internal standards was incubated at 37°C for 2 h at pH 5.2. After addition of 25 μl of Tris-HCl (1 M, pH 9.0) and 70 U of alkaline phosphatase, the sample was incubated for an additional 2 h at pH 8.2. Samples were analyzed using liquid chromatography tandem mass spectrometry (LC-MS/MS). The methodology and internal standards used for measuring oxidative DNA damage at the dimer level have been described previously [20-23].

Since the use of the d(P^f^pA) and d(T^g^pA) base modifications as measures of oxidatively generated DNA damage is relatively new, we examined repeatability of these measurements. Pairs of samples from the same participant were prepared and analyzed in parallel. The average values for the two set differed by 6% for the d(T^g^pA) modification (12 pairs; r = 0.89) and 5% for the d(P^f^pA) modification (36 pairs; r = 0.74).

### Data analysis

To assure a 'clean' sample for assessment of cessation effects on levels of oxidative damage, analyses were limited to those who reported complete cessation and demonstrated CO-confirmed abstinence at 4 weeks and 11 weeks after TQD. The initial approach to analysis was descriptive (Pearson correlations, cross tabulations, t-tests) and focused on patient demographics and base modifications across subjects and weeks. Generalized estimating equations (GEE) were employed to examine the significance of change in biomarkers across time, accounting for the within-subjects dependence of measurements and adjusting for gender, treatment group (Active, Placebo) and age [24]. The GEE models used a normal distribution with log link function, and an exchangeable working correlation matrix for the repeated measurement. Analyses were conduced using SPSS 16.0 (SPSS Inc., Chicago, IL).

## Results

Demographic and tobacco use history data among participants are summarized in Table 1. We examined baseline values for cigarette consumption, exhaled CO, and the three DNA lesions measured as a function of participant demographics. We observed higher baseline exhaled breath CO levels among male compared to female participants (48.0 ppm (SEM 5.1) vs. 33.1 ppm (SEM 4.3)), and also noted a significant correlation between cigarettes smoked per week and CO levels. At baseline there was no significant relationship among these metrics and the three DNA lesions of interest.

**Table 1:** Demographic and smoking behavior characteristics of the sample and interrelationships among measures at baseline

				t-statistic and p-value for comparison on demographic variables				
		**N (%)**		**Cigs**	**CO**	**d(T**^**g**^**pA)**	**d(P**^**f**^**pA)**	**dG**^**h**^

Gender	Male	10 (52%)	*t*	1.187	**2.205**	-0.409	-0.318	-0.580
						
	Female	9 (48%)	*p*	0.251	**0.041**	0.688	0.755	0.569

Race	White	15 (79%)	*t*	1.259	0.434	-0.849	-0.647	1.145
						
	Black	4 (21%)	*p*	0.225	0.670	0.408	0.526	0.268

Treatment Group	Active	13 (68%)	*t*	-0.577	0.109	-0.163	0.845	1.688
							
	Placebo	6 (32%)	*p*	0.571	0.914	0.873	0.410	0.110

		Mean (SD)		Pearson correlation and p-value between biomarkers and smoking variables				

Age (years)		50.2 (10.1)	*r*	-0.12	0.31	-0.03	-0.10	-0.35
			p	0.630	0.199	0.889	0.680	0.146

Years Smoked		28.8 (9.6)	*r*	0.22	0.40	0.04	0.01	-0.14
			*p*	0.358	0.090	0.861	0.972	0.573

Cigarettes per Week		126 (33)	*r*	1.00	**0.56**	-0.09	-0.18	0.04
			*p*	--	**0.013**	0.706	0.474	0.881

Carbon Monoxide (ppm)		40.9 (3.7)	*r*	**0.56**	1.00	-0.29	-0.22	-0.05
			*p*	**0.013**	--	0.225	0.357	0.848

Statistically significant effects noted in boldface.

Cigs = Cigarettes smoked per week (N)

CO = Carbon monoxide (ppm)

d(T^g^pA) = glycol modification of thymine (fmol/μg)

d(P^f^pA) = formamide breakdown product of pyrimidine bases (fmol/μg)

d(G^h^) = 8-oxo-7,8-dihydroguanine (fmol/μg)

**Figure 3:**
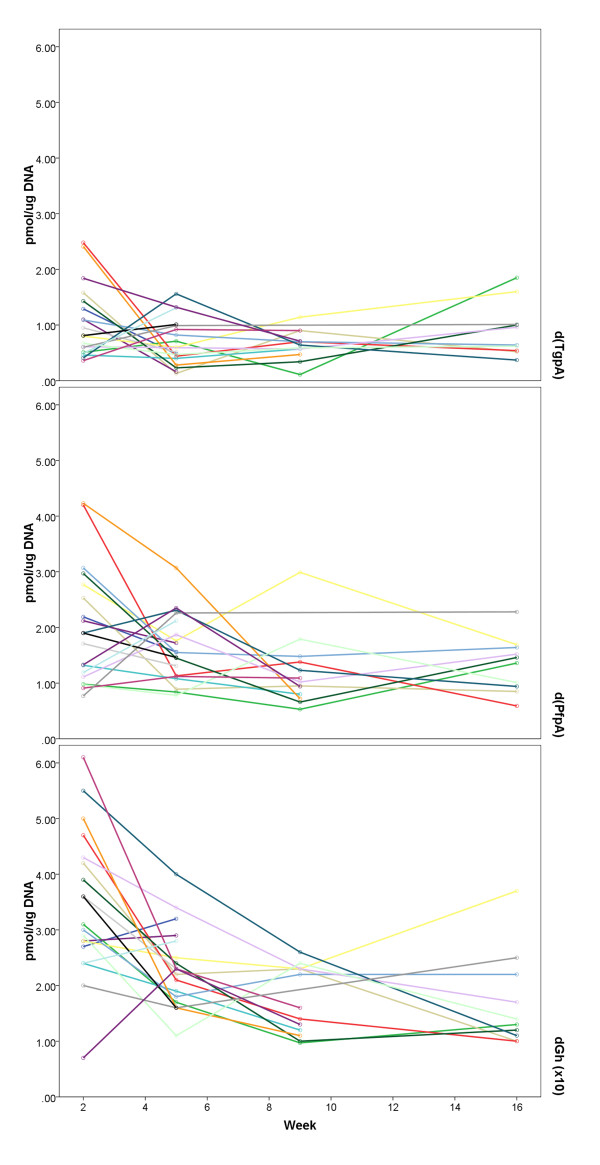
**Individual patterns of change in lesion levels by study week**.

The results of measurements of d(T^g^pA), d(P^f^pA), and d(G^h^) modifications are presented in Table 2 for each of the four time points. The mean values of the measurements and SEM's are given in terms of femtomoles (fmol) of lesions per microgram (μg) of DNA. Figure 3 illustrates the interindividual variability in measurements at each timepoint as well as changes across time. An overall pattern was apparent for all 3 markers demonstrating decreases over time coinciding with stopping smoking. Two biomarkers (d(T^g^pA) and d(P^f^pA)) increased on average at the final measurement. Results of generalized estimating equation analysis (accounting for gender, age, and treatment condition; see Table 3) showed significant overall time effects for the d(T^g^pA) (χ^2^(3) = 9.389, p < 0.025), d(P^f^pA) (χ^2^(3) = 9.070, p < 0.028), and d(G^h^) (χ^2^(3) = 37.236, p < 0.001) lesions, indicating levels of each decreased significantly after smoking cessation. The d(T^g^pA) and d(P^f^pA) lesions show relatively greater rebound at TQD+11 weeks compared to the d(G^h^) lesion (88% of baseline for d(T^g^pA), 64% of baseline for d(P^f^pA), vs 46% of baseline for d(G^h^). Indeed, the GEE models show that the value 11 weeks following TQD is not statistically different from the baseline value for d(T^g^pA), however those differences are significant for d(P^f^pA) and d(G^h^). We did not see a significant association of lesion levels with age or gender. In the case of the d(G^h^) lesion, we observed a statistically significant treatment group difference (χ^2^(1) = 4.910, p < 0.027), wherein those in the treatment arm had overall significantly higher d(G^h^) levels (23.0 versus 18.1).

**Table 2:** Geometric mean thymine glycol (d(T^g^pA)), formamide (d(P^f^pA)) and 8-oxo-7,8-dihydroguanine (dG^h^) lesions (fmol/μg) by week, unadjusted and adjusted for age, gender, and treatment condition

Sample (N)	% abstinent	Cigarette per week (SEM)	**d(T**^**g**^**pA) fmol/μg (SEM)**		**d(P**^**f**^**pA) fmol/μg (SEM)**		**dG**^**h **^**fmol/μg (SEM)**	
			**Unadj**	**Adj**	**Unadj**	**Adj**	**Unadj**	**Adj**

TQD - 4 weeks [baseline] (N = 19)	0	126 (7.5)	0.89 (1.15)	1.06 (0.15)	1.77 (1.13)	1.93 (0.20)	31.70 (1.12)	33.15 (2.49)

TQD [week 0] (N = 19)	68.4	85 (8.7)	0.55 (1.19)	0.71 (0.10)	1.51 (1.09)	1.55 (0.12)	21.97 (1.07)	21.48 (1.68)

TQD + 4 weeks [week 4] (N = 13)	100	0	0.57 (1.17)	0.64 (0.09)	1.07 (1.14)	1.12 (0.17)	16.40 (1.11)	15.76 (1.65)

TQD +11 [week 11] (N = 10)	100	0	0.81 (1.18)	0.91 (0.13)	1.25 (1.13)	1.23 (0.14)	15.56 (1.15)	15.43 (2.60)

NOTE: Abstinence defined by exhaled breath CO ≤ 8 ppm. Adjusted means are from GEE model including only those cases reporting no cigarettes smoked in the past week and showing CO-confirmed abstinence and controlling for treatment condition, gender, and age. TQD, target quit date.

**Table 3:** Parameter estimates from GEE models for each modification (statistically significant beta weights highlighted in bold)

	**d(T**^**g**^**pA)**				**d(P**^**f**^**pA)**				**dG**^**h**^			
	**B**	**SE**	**Wald χ**^**2 **^**(df = 1)**	**p**	**B**	**SE**	**Wald χ**^**2 **^**(df = 1)**	**p**	**B**	**SE**	**Wald χ**^**2 **^**(df = 1)**	p

Intercept	0.015	0.52	0.001	0.078	0.486	0.487	0.993	0.319	3.815	0.208	336.884	<0.001

Active Tx	REF				REF				REF			

Placebo	0.044	0.11	0.020	0.185	-0.193	0.132	2.139	0.085	**-0.244**	**0.110**	**4.910**	**0.027**

Female	REF				REF				REF			

Male	0.145	0.11	1.758	0.185	0.120	0.141	0.730	0.393	0.083	0.097	0.733	0.392

TQD - 4 weeks [baseline]	REF				REF				REF			

TQD [week 0]	-0.398	0.22	3.170	0.075	-0.221	0.129	2.958	0.085	**-0.434**	**0.094**	**21.303**	**<0.001**

TQD + 4 weeks [week 4]	**-0.496**	**0.17**	**8.454**	**0.004**	**-0.546**	**0.191**	**8.196**	**0.004**	**-0.744**	**0.124**	**36.068**	**<0.001**

TQD +11 [week 11]	-0.147	0.24	0.387	0.534	**-0.447**	**0.187**	**5.704**	**0.017**	**-0.765**	**0.202**	**14.299**	**<0.001**

Age	-0.001	0.008	0.020	0.889	0.004	0.009	0.229	0.632	-0.005	0.004	1.549	0.213

## Discussion

The findings from this study indicate that cigarette smoking appears to be related to oxidatively generated DNA damage, and that smoking cessation may reduce levels of oxidative damage between 30-50 percent in the short term (11 weeks following cessation). This study supports earlier findings [13,14] that the d(G^h^) lesion is associated with smoking, and also supports findings by Nia and colleagues [15] which reported decreases in d(G^h^) after smoking cessation. This study also provides preliminary evidence that smoking may also contribute to formamide [d(P^f^pA)] DNA lesions, while evidence for a relationship between smoking and the thymine glycol [d(T^g^pA)] lesion was weaker. Carmella and colleagues [25] proposed that sensitivity to changes in smoking (e.g., cessation) within-subjects is a strong indicator of the utility of a biomarker, as it allows individuals to serve as their own controls, minimizing the potential role for individual differences in DNA repair or toxicant metabolism as possible explanations for observed variations. By this standard, d(G^h^) and d(P^f^pA) may be promising biomarkers for future evaluation.

We are unable to explain the slight increase in levels of two biomarkers at the final measurement, despite controlling for CO-confirmed abstinence and self-reported cigarette use. One hypothesis is that these markers are sensitive to small exposures to cigarette smoke, including exposure to secondhand smoke (which was not measured) as well as other environmental exposures/sources. Indeed, other research has noted exposure to SHS as a potential source of oxidative damage in nonsmokers, [17,18] so this remains a plausible explanation. Alternatively, it may reflect the normal range of variability in oxidative damage with an individual over time.

A difficulty associated with assessing oxidative DNA damage caused by a single mechanism, such as smoking, is that a substantial level of damage is always present. Further, other environmental and demographic factors, particularly age, gender and diet, may influence damage levels. The contribution of smoking to oxidative DNA damage must be assessed by the incremental change produced by the behavior relative to background levels. A significant advantage of the present study was the longitudinal assessment of DNA damage among a group of smokers who participated in a smoking cessation trial; with this design individuals served as their own controls. However, this study also had weaknesses, including a small sample size, examination of only three DNA modifications, and lack of data regarding other sources of oxidative stress. For example, smoking appears to influence forms of oxidative stress beyond oxidatively induced DNA damage, such as lipid peroxidation [26]. Recent papers have used other approaches and markers of oxidative stress related to cigarette smoking in addition to d(G^h^), including isoprostanes, hydroxyeicosatetraenoic acid products (HETEs), and advanced glycation end-products [27-29]. Future research should look to examine the contribution of smoking to oxidative stress in a broader context, including additional markers, other sources of damage, and individual DNA repair capacity.

Overall, results from this analysis suggest that cigarette smoking contributes to the burden of oxidative DNA damage in smokers, but that the level of such DNA modifications may be reduced by stopping smoking. Future research may shed light on the broader array of oxidative damage influenced by smoking, to provide further insights into mechanisms underlying carcinogenesis.

## Acronyms/Abbreviations

CO: Carbon monoxide; d(G^h^): 8-oxo-7,8-dihydroguanine; d(P^f^pA): formamide breakdown product of pyrimidine bases; d(T^g^pA): glycol modification of thymine; GEE: Generalized estimating equations; LC-MS/MS: liquid chromatography tandem mass spectrometry; TQD: Target quit day

## Competing interests

The authors declare that they have no competing interests.

## Authors' contributions

HCB, RJO, KMC, and MCM conceived the study. HBP, HI, JBD, HGF, and EEB performed the analytical work. RJO conducted the statistical analysis. All authors contributed to manuscript preparation and approved the final draft.
